# Sleep-Related Cognitive/Behavioral Predictors of Sleep Quality and Relapse in Individuals with Alcohol Use Disorder

**DOI:** 10.1007/s12529-020-09901-9

**Published:** 2020-05-27

**Authors:** Alyssa Todaro Brooks, Narjis Kazmi, Li Yang, Ralph Thadeus Tuason, Michael Charles Krumlauf, Gwenyth Reid Wallen

**Affiliations:** Nursing Research and Translational Science, National Institutes of Health Clinical Center, 10 Center Drive Room 2B13, Bethesda, MD 20892 USA

**Keywords:** Alcohol use disorder, Insomnia, Sleep disturbance, Cognitive behavioral therapy for insomnia, alcohol, sleep

## Abstract

**Background:**

Little is known about cognitive and behavioral predictors of sleep quality and relapse among individuals with alcohol use disorder (AUD). Using the social cognitive theory (SCT), we assessed sleep-related behaviors and cognitions, sleep quality, and relapse to drinking among individuals with AUD transitioning from inpatient to outpatient settings.

**Method:**

Individuals (*n* = 149) seeking treatment for AUD were recruited during their inpatient stay. Self-efficacy for sleep, dysfunctional beliefs about sleep, sleep-related behaviors, sleep quality, and relapse were assessed. Objective (actigraphy) assessment of sleep efficiency and duration was measured using actigraphy. Multiple logistic regression models tested whether self-reported sleep quality or sleep-related beliefs/behavior predicted relapse. Repeated measures linear mixed modeling tested whether there was a change over time in sleep quality as well as the relationships between self-efficacy, sleep-related beliefs, sleep behaviors, sleep quality, and relapse.

**Results:**

In our sample, self-efficacy for sleep, dysfunctional beliefs about sleep, and sleep-related behavior were all significantly associated with both sleep quality and relapse. Controlling for pre-discharge sleep-related behaviors (SRBQ) and actigraphy-recorded average sleep time during the first week post-discharge, married participants had lower odds of relapse compared with non-married patients (*p* = 0.048, OR = 0.119, 95% CI 0.015–0.983). Patients with lower self-efficacy for sleep (SES) scores (*p* < 0.001) and higher CPRS anxiety scores (*p* < 0.001) had higher PSQI scores.

**Conclusion:**

Our results highlight the importance of self-efficacy and dysfunctional beliefs about sleep as predictors of sleep quality and relapse among individuals with AUD and the utility of the SCT as a sleep research framework.

**Electronic supplementary material:**

The online version of this article (10.1007/s12529-020-09901-9) contains supplementary material, which is available to authorized users.

## Background

Alcohol use disorder (AUD; formerly alcohol “abuse” or “dependence”) is often accompanied by various psychiatric and socio-behavioral comorbidities, including significant sleep disturbances [1–3]. Alcohol can negatively affect many aspects of sleep including the proportion of rapid eye movement (REM) sleep, nightmare frequency, sleep fragmentation, and snoring. These effects manifest in varying intensity through stages of drinking, withdrawal, and abstinence [4, 5]. Multiple studies have reported a prevalence of insomnia symptoms ranging from 36 to 91% among patients with alcohol dependence [6]. Ford and Kamerow [7] demonstrated that individuals who met criteria for alcohol abuse and dependence were more likely to report *ever* experiencing a period of two or more weeks of insomnia when compared with non-alcohol-dependent individuals.

Individuals may use alcohol to self-medicate sleep disturbance. Those with sleep disturbances may choose to drink alcohol specifically because of its depressive effects [8], and individuals with insomnia may be more likely to self-medicate with alcohol [9]. Heavy alcohol consumption can induce fatigue and reduce sleep onset latency thereby speeding up the process of falling asleep [10–12], which may be particularly tempting for those who are struggling with difficulty sleeping and already accustomed to drinking heavily. Recently, Roehrs and Thomas [13] uncovered the risk associated with using alcohol as a sleep aid; as tolerance develops, this results in self-administration of significantly higher amounts of alcohol for sleep. Specifically among alcohol-dependent individuals, re-initiation of drinking following abstinence may be an attempt to self-medicate sleep problems [12, 14, 15]. Kolla and colleagues [14] reported that in a sample of individuals who were alcohol dependent admitted for a 30-day addiction treatment program, more than half (51%) self-reported the use of alcohol to help them fall asleep and this use of alcohol significantly predicted relapse over a period of 12 months. Similarly, in another mixed methods study assessing sleep-related beliefs and behaviors of treatment-seeking AUD patients, participants reported the use of alcohol to self-medicate for sleep and anxiety [16].

Sleep disturbance may be associated with relapse to drinking. Among individuals who seek treatment, baseline sleep problems upon entering treatment may predict subsequent relapse to drinking [17–21]. Smith and colleagues [20] demonstrated that longer sleep onset latency during inpatient rehabilitation predicted relapse within one month of discharge from the treatment facility. Self-reported use of alcohol to fall asleep is associated with relapse over 12 months following a one-month residential treatment [14]. Individuals who report insomnia within six months prior to achieving abstinence are more likely to relapse after five months of abstinence [22].

### Theoretical framework

Both AUD and sleep disturbances are complex, multi-factorial, and impact multiple facets of individuals’ lives. We chose to use the social cognitive theory (SCT) to examine the relationship between sleep and relapse. The SCT has been widely used to predict and modify behavior, including abstinence [23]. The SCT posits that personal factors, the environment, and human behavior exert influence upon each other through *reciprocal determinism* [24]. A recent review called for theoretical frameworks to be used when assessing sleep, in order to better understand behavior [25]. Sleep-related cognitions, thoughts, and perceptions and sleep-related behaviors should be carefully considered in maximizing the success of recovery efforts. Unhealthy sleep-related cognitions are established contributors to poor sleep [26, 27]. Cognitive arousal and inaccurate beliefs about sleep can lead to maladaptive sleep behaviors [27]. Behaviors such as late-night physical activity, daytime napping, and sleeping in on weekends can be harmful to overall sleep quality [27]. Sleep-related expectations and behavior are both influenced by an individual’s sleeping *environment*: bedtime, lighting, temperature, pressure to attend to other obligations, bed-partner snoring, pets in the bedroom, and other factors. Lastly, a stronger belief in one’s ability to achieve better sleep (“self-efficacy for sleep”) may determine whether the response is a harmful sleep behavior or a healthier adaptation [24]. Preliminary findings in a small cohort (*n* = 95) of the current study by Brooks and colleagues [16] showed significant improvements in self-efficacy for sleep over the course of inpatient treatment through discharge. Despite potential relevance for assessing/improving sleep health, the SCT (in its entirety) has “been applied to sleep research only in the context of adherence to medical sleep disorder treatments” [25](p. 7) and thus represents a novel approach to examining sleep-related outcomes. Considering the importance of self-efficacy, outcome expectancies, and environment during transition from treatment in AUD as it relates to sleep, the SCT provided our theoretical framework to evaluate the potential relationships between sleep and relapse proposed in this study.

### Purpose of study

Little is known about cognitive and behavioral predictors of sleep quality and relapse among individuals with AUD. In the present study, we assessed sleep-related behaviors and cognitions, sleep quality (subjective and objective [actigraphy]), and relapse to drinking among individuals with AUD across the transition from an inpatient to outpatient setting. We also assessed basic demographic factors, including marital status, which could have implications for post-discharge environment/social support [28]. Our primary outcomes included sleep quality and relapse. We hypothesized the following: (1) higher self-efficacy for sleep would be associated with better sleep quality and lower relapse rates; (2) fewer dysfunctional beliefs about sleep would be associated with better sleep quality and lower relapse rates; (3) higher endorsement of sleep-related safety behaviors would be associated with poorer sleep quality and higher relapse rates; and (4) better sleep quality would be associated with lower relapse rates.

## Methods

We recruited individuals (*n* = 149) seeking inpatient treatment for AUD initially enrolled on a screening and assessment clinical trial (NCT#02231840). All participants received continued physical evaluations, inpatient treatment of alcohol withdrawal, psychosocial management, and an educational treatment program during their participation in this study. Participants were eligible for our study examining sleep disturbance throughout rehabilitation (NCT#02181569) if they were 18 years of age or older, an inpatient for 21 days or more preceding discharge, not enrolled onto a pharmacologic intervention study, able to understand the study, and willing to complete a follow-up visit in-person or by phone four to six weeks after being discharged from inpatient treatment. The analysis described herein represents a sub-analysis of the total sample of 198 participants and includes only individuals who provided data on sleep quality/relapse post-discharge. All participants provided written informed consent.

We used basic demographic, clinical, and alcohol-related variables to characterize the sample upon admission, including age, gender, marital status, race, ethnicity, the Structured Clinical Interview for DSM IV or DSM 5 diagnosis (SCID; [29]), alcohol drinking history before admission using Timeline Follow-back (TLFB; [30, 31]), alcohol craving using the Penn Alcohol Craving Scale (PACS; [32]), severity of alcohol withdrawal using the Clinical Institute Withdrawal Assessment (CIWA; [33]), and depression and anxiety using two subscales of the Comprehensive Psychopathological Rating Scale (CPRS; [34–36]); brief scale for anxiety (BSA) and Montgomery-Asberg depression rating scale (MADRS).

Our primary outcome of sleep quality was self-reported (Pittsburgh Sleep Quality Index-PSQI; [37–39]). We also assessed excessive daytime sleepiness (Epworth Sleepiness Scale-ESS; [40–43]), dysfunctional beliefs/attitudes about sleep (Dysfunctional Beliefs and Attitudes about Sleep Scale, DBAS-16; [44]), self-efficacy for sleep (Self-Efficacy for Sleep Scale, SE-S; [45–47]), and sleep-related behaviors (Sleep-Related Behaviors Questionnaire, SRBQ; [48]). Refer to Supplemental Table 1 for a description of all measures and the timing of their administration.

### Objective measure of sleep: Respironics Actiwatch Spectrum Plus

“Actiwatches” are small actigraphy-based wristband data loggers that record digitally integrated measures of gross motor activity. These devices contain accelerometers and light sensors in order to objectively assess sleep and activity. Prior studies have demonstrated actigraphy’s high sensitivity with moderate accuracy for assessing sleep parameters in populations with normal and disturbed sleep when compared with polysomnography [49–51]. The *Actiwatch Spectrum Plus* provided a battery life adequate for the outpatient research phase of this protocol. The watch is worn on the non-dominant wrist. For approximately one week before their scheduled discharge date, patients wore an Actiwatch Spectrum *Plus* (Philips Respironics) up until four weeks from their date of discharge, which coincided with the final visit for follow-up surveys. Main outcomes from actigraphy included weekly averages of sleep efficiency, wake after sleep onset, and time in bed.

### Sleep/relapse diary

We used daily sleep and symptom diaries to cross-validate objective sleep data collected via actigraphy and assess additional symptoms not captured by other assessments. We assessed relapse from patient report of alcohol consumption in the diary (primary outcome) and asked patients to add up the number of alcoholic drinks consumed per day. Patients were instructed to complete diaries once daily (in the morning upon waking).

### Study timeline overview

Approximately one week prior to patients’ scheduled discharge, a study team member approached patients to begin the first segment of data collection for the study. Participants completed assessments of daytime sleepiness, self-efficacy for sleep, and sleep-related beliefs and behaviors at this time. Four to six weeks following discharge, participants completed a follow-up visit in-person or by phone.

### Actigraphy analyses

After device removal and data download, raw data from the *Actiwatch Spectrum Plus* were analyzed using the *Respironics* computerized sleep scoring software, which scores each epoch based on a threshold method algorithm. Investigators reviewed each sleep period prior to analysis to screen for malfunctioning watches, corrupt data, and required adjustments using bedtimes and wake times from the diary self-reports when necessary.

### Statistical analysis

All data were double-data entered, cross-checked, and reconciled as necessary. We dichotomized marital status (married vs. non-married) and used the last possible CPRS scores prior to discharge (day 23 or 30 depending on patients’ length of stay). For withdrawal scores, we used the overall maximum score of any day of treatment. We treated relapse as a dichotomous variable; *any* drinking within four weeks of discharge from inpatient treatment was considered relapse based on diary self-report. Relationships between variables were examined with bivariate correlation coefficients (for two continuous variables), chi-squares (for two categorical variables), and basic *t* tests (for one categorical and one continuous variable). All variables associated with relapse with *p* values less than 0.20 were included in the multiple logistic regression model to test whether sleep quality or sleep-related beliefs/behavior predict relapse. Backward stepwise with an entering criterion of 0.05 and a removal criterion of 0.10 to select the variables in the final model was used. Repeated measures linear mixed modeling was performed to test whether there was a change over time in sleep quality as well as the relationships between sleep quality and relapse, self-efficacy, sleep-related beliefs, and sleep behaviors. A restricted maximum likelihood (REML) procedure for model parameter estimation was utilized. We used Aikake information criterion (AIC) and Bayesian information criterion (BIC) to compare and select models. All data analyses were conducted using IBM SPSS statistics and SAS 9.4. A *p* value less than 0.05 was considered statistically significant.

## Results

Study participants (*n* = 149) were mostly male (*n* = 99, 66.4%) and not married (*n* = 119, 79.9%). Of the 119 unmarried individuals, 21 (14.1%) were divorced, seven (4.7%) were separated, 88 (59.1%) were single, three (2.0%) were widowed, and three (2.0%) did not report a marital status. We compared demographic and clinical variables between those who relapsed and those who did not relapse (Table 1 and Table 2). Those who relapsed were younger (44.97 ± 12.75 vs. 49.59 ± 10.18, *p* = 0.045), less likely to be married (3.3% vs. 26.4%, *p* = 0.004), more likely to report higher levels of post-discharge craving (PACS score of 13.04 ± 7.62 vs. 7.11 ± 6.08, *p* < 0.01), more likely to self-report sleep disturbance pre-discharge (mean PSQI score of 9.0 ± 3.10 vs. 7.27 ± 4.11, *p* = 0.017), had lower self-efficacy for sleep post-discharge (26.32 ± 7.05 vs. 29.89 ± 7.59, *p* = 0.035), self-reported more dysfunctional beliefs about sleep before discharge (4.70 ± 1.68 vs. 3.81 ± 1.96, *p* = 0.027), and engaged in more sleep-related behaviors (SRBQ score 52.73 ± 14.22 vs. 43.66 ± 15.73 *p* < 0.01 and 51.36 ± 20.21 vs. 42.14 ± 19.03, *p* = 0.04 respectively) at both time points. For both individuals who relapsed and those who did not relapse, participants’ weekly average sleep duration improved from week 1 to week 4 (Table 2), but there were no significant differences between relapse status groups on any actigraphy variables at either time point.

**Table 1:** Demographic and clinical variables by relapse groups (*N* = 149)

Demographics *n* = 149	Total sample *n* = 149	Relapse *n* = 30	No relapse *n* = 93	*p* value
Age, mean (SD)	47.30 (11.64)	44.97 (12.75)	49.59 (10.18)	0.045
Gender, *n* (%)				0.27
Male	99 (66.4)	17 (56.67)	64 (68.82)	
Female	50 (33.6)	13 (43.33)	29 (31.18)	
Marital status				0.004
Married	27 (18.5)	1 (3.3)	24 (26.4)	
Single/widowed/divorced/separated	119 (79.9)	29 (96.7)	67 (73.6)	
Race (%)				0.239
White	81 (54.4)	17 (56.67)	52 (55.91)	
Black	51 (34.2)	7 (23.33)	32 (34.41)	
Other	17 (11.4)	6 (20.0)	9 (9.68)	
Ethnicity (%)				0.308
Not Hispanic or Latino	137 (91.9)	26 (86.7)	86 (93.5)	
Hispanic or Latino	10 (6.7)	4 (13.3)	5 (5.4)	
Unknown	1 (0.7)	0	1 (1.1)	
SCID-IV/5^a^, *n* = 148				
One or more anxiety disorders (%)	44 (29.53)	11 (36.7)	26 (28.3)	0.493
One or more mood disorders (%)	45 (29.53)	11 (36.7)	27 (29.3)	0.499
Timeline Follow-back (90 days preceding admission) *N* = 145 Mean (SD)				
Average drinks/day, *n* = 145	15.31 (8.48)	15.24 (8.17)	15.56 (8.52)	0.857
Number of drinking days, *n* = 145	73.74 (23.26)	78.27 (16.85)	73.04 (25.67)	0.206
Number of heavy drinking days, *n* = 145	70.32 (26.05)	75.70 (21.77)	68.99 (28.59)	0.183
Penn Alcohol Craving Scale Mean (SD)				
Day 5 of inpatient admission, *n* = 147	10.74 (7.37)	11.40 (7.59)	10.82 (7.49)	0.712
Post-discharge, *n* = 111	8.35 (6.91)	13.04 (7.62)	7.11 (6.08)	0.000
Clinical Institute Withdrawal Assessment (CIWA) Max score mean (SD)	8.52 (5.96)	10.20 (8.11)	8.11 (4.84)	0.19
Comprehensive Psychopathology Rating Scale (CPRS)^b^				
Brief Scale for Anxiety (BSA), mean (SD)				
Day 2 of inpatient admission, *n* = 148	12.72 (6.69)	14.28 (5.98)	12.0 (6.77)	0.107
Pre-discharge, *n* = 149	5.22 (4.70)	5.90 (4.75)	5.12 (4.63)	0.426
Montgomery–Åsberg Depression Rating Scale (MADRS) mean (SD)				
Day 2 of inpatient admission, *n* = 148	16.72 (8.61)	18.83 (8.90)	16.01 (8.60)	0.129
Pre-discharge, *n* = 149	5.21 (5.52)	6.37 (6.12)	5.22 (5.53)	0.336

^a^82 participants were assessed with the DSM SCID-IV and 66 were assessed with DSM SCID-5

^b^Depending on participants’ length of stay, last available scores before discharge were used for pre-discharge BSA and MADRS; *n* = 99 from day 30 and *n* = 50 from day 23

**Table 2:** Sleep measures by relapse status (*N* = 149)

Sleep measures Mean (SD)	Total sample *n* = 149	Relapse *n* = 30	No relapse *n* = 93	*p* value*
Pittsburgh Sleep Quality Index (PSQI)				
Pre-discharge, *n* = 148	7.74 (3.93)	9.00 (3.10)	7.27 (4.11)	0.017
Post-discharge, *n* = 109	7.28 (3.85)	8.64 (3.19)	7.03 (3.98)	0.067
Self-Efficacy for Sleep (SE-S)				
Pre-discharge, *n* = 148	28.55 (6.66)	27.23 (5.22)	28.83 (7.09)	0.19
Post-discharge, *n* = 111	29.19 (7.67)	26.32 (7.05)	29.89 (7.59)	0.035
Dysfunctional Beliefs and attitudes about Sleep (DBAS)				
Pre-discharge, *n* = 149	3.96 (1.98)	4.70 (1.68)	3.81 (1.96)	0.027
Post-discharge, *n* = 111	3.81 (2.10)	4.02 (2.02)	3.86 (2.12)	0.739
Epworth Sleepiness Scale (ESS)				
Pre-discharge, *n* = 149	6.33 (4.05)	6.53 (4.02)	5.89 (4.16)	0.455
Post-discharge, *n* = 111	5.59 (4.03)	5.16 (3.60)	5.78 (4.18)	0.507
Sleep-Related Behaviors Questionnaire (SRBQ)				
Baseline/pre-discharge *n* = 149	45.01 (16.56)	52.73 (14.22)	43.66 (15.73)	0.006
4 weeks post-discharge *n* = 111	43.44 (19.83)	51.36 (20.21)	42.14 (19.03)	0.039
Actigraphy variables				
Sleep duration				
Baseline week 1 *n* = 120	433.59 (89.87)	411.51 (107.50)	438.40 (84.60)	0.173
Week 4 post-discharge *n* = 106	442.19 (91.73)	425.64 (99.27)	447.29 (89.22)	0.229
Sleep efficiency				
Baseline week 1 *n* = 120	80.22 (8.18)	78.54 (9.68)	81.13 (7.45)	0.141
Week 4 Post-discharge *n* = 106	80.22 (9.23)	80.99 (11.86)	80.08 (8.41)	0.669
Wake after sleep onset (WASO) minutes				
Baseline week 1 *n* = 120	60.05 (21.90)	61.38 (20.35)	57.55 (20.10)	0.381
Week 4 post-discharge *n* = 106	61.37 (29.01)	53.73 (23.19)	63.26 (29.16)	0.139
Sleep onset latency				
Baseline week 1 *n* = 120	15.78 (16.43)	14.03 (16.72)	16.06 (16.63)	0.574
Week 4 post-discharge *n* = 106	18.22 (19.17)	14.74 (16.31)	18.92 (19.63)	0.338
Total sleep time				
Baseline week 1 *n* = 120	372.63 (84.43)	348.84 (102.42)	380.11 (77.90)	0.09
Week 4 post-discharge *n* = 106	379.87 (83.21)	371.01 (100.91)	383.04 (78.19)	0.589

*p values reflect comparison between relapse groups

**Fig. 1:**
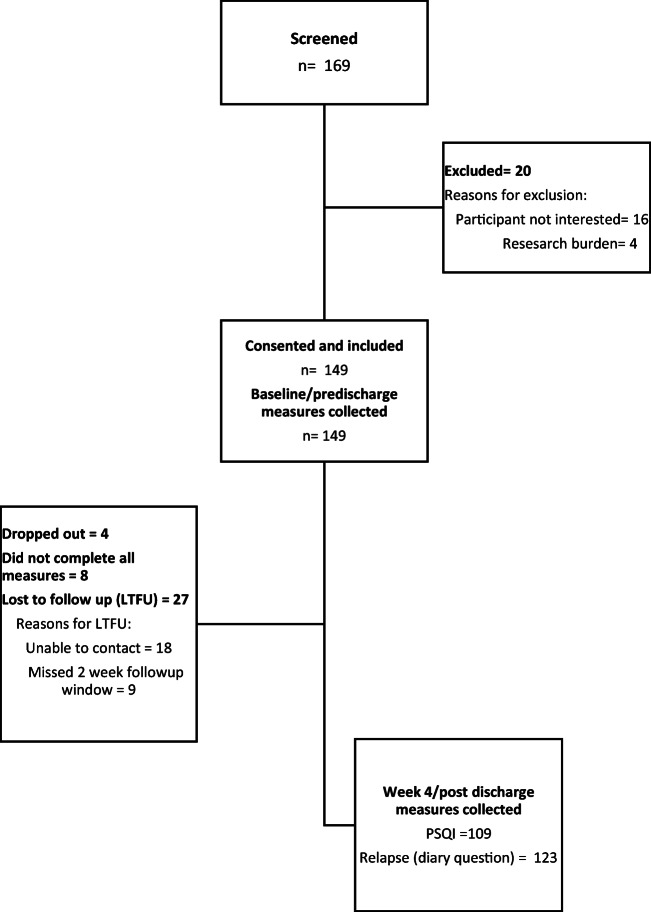
Flow diagram outlining participant recruitment and retention

Refer to Fig. 1 for a flow diagram outlining participant recruitment and retention. Since the two primary outcomes included sleep quality (PSQI) and relapse, we assessed differences between those who completed each of the aforementioned assessments and those who did not. There were no significant gender, race, ethnicity, or marital status differences between those who provided (*n* = 123) and who did not provide (*n* = 26) relapse data. Those who did not provide relapse data for 28 days post-discharge were significantly younger (41.8 vs. 48.5, *p* = 0.007). There were no significant differences between those who provided (*n* = 109) and who did not provide (*n* = 40) PSQI data. Those who did not provide PSQI data 4–6 weeks post-discharge had significantly higher actigraphy-recorded mean wake bouts during week 1 (28.9 vs. 24.3, *p* = 0.017).

### Results of hypothesis testing. Hypothesis 1

Higher self-efficacy for sleep (SE-S) is associated with better sleep quality and lower relapse rates. For both time points, higher SE-S was associated with a lower PSQI score (*p* < 0.001). No significant differences were found in pre-discharge self-efficacy scores between participants who relapsed (27.23 ± 5.22) and participants who did not relapse (28.83 ± 7.09, *p* = 0.19).

### Hypothesis 2

Fewer dysfunctional beliefs and attitudes about sleep (DBAS) was associated with better sleep quality and lower relapse rates. For both time points, a higher DBAS score was associated with higher PSQI scores (*p* < 0.001). Patients who relapsed had significantly higher week 1 DBAS scores (4.70 ± 1.68 vs. 3.81 ± 1.96, *p* = 0.027).

### Hypothesis 3

Higher endorsement of sleep-related safety behaviors (SRBQ) is associated with poorer sleep quality and higher relapse rates. For both time points, higher SRBQ scores were associated with higher PSQI scores (*p* < 0.001). Patients who relapsed had significantly higher week 1 SRBQ scores (52.73 ± 14.22 vs. 43.66 ± 15.73, *p* = 0.006).

### Hypothesis 4

Better sleep quality is associated with lower relapse rates. Individuals who relapsed had higher pre-discharge PSQI scores (9.0 ± 3.1 vs. 7.27 ± 4.11, *p* = 0.017).

### Model 1: relapse

In the final model of relapse (Table 3), controlling for pre-discharge sleep-related behaviors (SRBQ) and actigraphy-recorded average sleep time during the first week post-discharge, married patients had lower odds of relapse compared with non-married patients (*p* = 0.048, OR = 0.119, 95% CI 0.015–0.983). Controlling for marital status and actigraphy-recorded average sleep time during the first week post-discharge, patients with higher sleep-related behaviors scores had higher odds of relapse (*p* = 0.026, OR = 1.046, 95% CI 1.006–1.088).

**Table 3:** Predictors of relapse

Predictors	Beta	Std. Error	Wald-statistic	95% CI	*p* value
Married	− 2.125	1.075	3.904	0.015–0.983	0.048
Sleep-related behaviors (SRBQ)^a^	0.045	0.020	4.987	1.006–1.088	0.026
Total sleep time^b^	− 0.006	0.003	3.744	0.987–1.000	0.053

Variable(s) entered in the logistic regression model were age, married, baseline/pre-discharge DBAS, PSQI, SRBQ, sleep efficiency, sleep time, SE-S, post-discharge PSQI, and MAX CIWA days 1–4

^a^Baseline/pre-discharge data

^b^Average from week 1 (post-discharge) of actigraphy

### Model 2: sleep quality (PSQI)

There were no significant changes in PSQI scores from pre- to post-discharge. In the model predicting sleep quality (Table 4), controlling for all other variables in the model, patients with lower self-efficacy for sleep (SES) scores (*p* < 0.001) and higher pre-discharge CPRS anxiety scores (*p* < 0.001) had higher PSQI scores.

**Table 4:** Predictors of sleep quality

Predictors	Estimate	Std. Error	*t*-statistic	95% CI	*p* value
No relapse	− 1.05	0.54	− 1.948	− 2.122 to 0.019	0.054
Dysfunctional Beliefs and Attitudes about Sleep (DBAS)^a^	0.23	0.12	1.857	− 0.014 to 0.470	0.065
Self-Efficacy for Sleep (SE-S)^a^	− 0.18	0.04	− 4.741	− 0.248 to 0.102	0.000
Sleep onset latency^a^	0.01	0.01	0.874	− 0.013 to 0.034	0.383
Max CIWA days 1–4	0.06	0.04	1.595	− 0.015 to 0.146	0.114
CPRS anxiety pre-discharge	0.23	0.05	4.219	0.121 to 0.335	0.000

^a^Longitudinal data from both timepoints was entered in the model

## Discussion

Results of the current study highlight the importance of cognitive and behavioral predictors of sleep quality and relapse among individuals with AUD. In our sample, self-efficacy for sleep, dysfunctional beliefs about sleep, and sleep-related behavior were all significantly associated with both sleep quality and relapse. Additionally, sleep quality was associated with relapse. Similar to previous findings [17–21], our results further describe that among treatment-seeking individuals, baseline sleep disturbances at the start of transition from inpatient to outpatient environment may predict relapse to drinking. This evidence makes the case for improving sleep quality as an important target for comprehensive and targeted treatment among individuals with AUD.

The role of marital status in predicting relapse was an interesting finding and further supports our previous qualitative work [16] in the same population, which pointed to social support as a potentially important variable in sustained sobriety. These results support the need for future research that considers post-discharge social support more broadly. Understanding the immediate support network following inpatient treatment, including spouses and other family members as well as their drinking patterns and the type of support they provide, could help researchers and clinicians customize treatment plans.

In our sample, there were no statistically significant differences in actigraphy-recorded variables between those who relapsed and those who did not relapse. Instead, differences between the cognitive and behavioral variables (specifically self-efficacy for sleep, dysfunctional beliefs and attitudes about sleep, and sleep-related behaviors) were more pronounced between the two groups.

As in other studies [15, 52], we found that even in those who did not relapse, and whose sleep quality improved, sleep disturbance persisted post-abstinence. This persistence of sleep disturbances has important implications for how interventions such as cognitive behavioral therapy for insomnia (CBT-I) might be an important complement for therapy focused on sobriety [53]. Furthermore, our results emphasize the importance of improving self-efficacy related to sleep, addressing anxiety, and establishing healthy beliefs and behaviors related to sleep to improve sleep quality and increase the likelihood of sobriety.

### Strengths and limitations

The use of the SCT to ground our measures and analyses and identify cognitions/behaviors associated with sleep quality and relapse is a strength of this analysis; to our knowledge, it has not previously been used to explore sleep in populations with AUD. Although our analysis did not specifically measure reciprocal determinism through the interactions between self, behavior, and the environment, future studies in individuals with AUD should be designed to measure interventions that promote behavior change, including changes to optimize the environment and influence personal attitudes and beliefs about sleep. Additionally, the use of both objective (actigraphy) and subjective assessments of sleep is a strength. One methodological advantage was the use of sleep and symptom diaries to assess relapse. In our patient population, it is often difficult to obtain accurate information from the Timeline Follow-back assessment (post-discharge) for various reasons, including but not limited to losing patients to follow-up. For the participants who completed the study, the diaries provided a short-term mode of assessing relapse. Our study is not without limitations. A number of participants did not complete the outcome measures for various reasons, including not wanting to wear the Actiwatch and being lost to follow-up/missing the follow-up “window.” While much of the existing literature focuses on insomnia rates in populations with AUD, we did not measure insomnia and instead used a more broad measure of sleep disturbance (the PSQI). Our measure of “environment” (for sleep/recovery) was limited; although marital status emerged as an important factor in terms of relapse, it is not necessarily descriptive of the post-discharge environment (e.g., returning to a home, entering a structured living facility). We relied on self-report for assessing relapse, which is a limitation, but objective measures of relapse were beyond the scope of this study. Finally, the sample was not representative of all individuals with AUD seeking treatment and willing to participate in research; findings cannot be generalized.

## Conclusions/Future Directions

Sleep-related beliefs and behaviors as well as sleep quality are important components of health-related quality of life, but may be particularly important among individuals with AUD. This is especially true when considering how best to support patients in their efforts to abstain from drinking across the transition from an inpatient treatment program back home and beyond. Results of the current analysis provide support for the SCT as a conceptual model in assessing cognitive and behavioral predictors of sleep quality and relapse among individuals with AUD with specific focus on the constructs of self-efficacy, outcome expectancies, and environment. Future research should consider novel methods of assessing both sleep disturbance/insomnia and relapse to drinking, as well as longer periods of follow-up. In larger datasets, it may be beneficial to distinguish between total sobriety and *any* or *heavy* drinking (potentially even treating relapse as a continuous variable—i.e., total or average number of drinks consumed) to allow for more nuanced data analyses. Since marital status emerged as an important variable in our analysis, future studies could assess quality/level of support provided by spouses (and other sources) post-discharge, in order to better understand outcomes. Based on our previous work regarding the importance of social support and our current results showing marital status as a predictor of relapse, we are interested in examining the effects of structured living (i.e., Oxford Houses or the like) vs. returning to the same pre-treatment environment, with the ultimate goal of targeted interventions based on risk. Other aspects of the post-discharge environment may also play an important role and should be explored.

## Electronic Supplementary Material

ESM 1 (DOCX 32 kb)
