# Supplementary material for: Sleep-Related Cognitive/Behavioral Predictors of Sleep Quality and Relapse in Individuals with Alcohol Use Disorder
Source: Int J Behav Med. 2020 May 27;28(1):73–82. doi: 10.1007/s12529-020-09901-9 (PMC7925448; doi:10.1007/s12529-020-09901-9)
Supplement: Supplementary file 1 — (DOCX 32 kb) [file 12529_2020_9901_MOESM1_ESM.docx]

**Supplemental Table 1:** *Study Measures*

| Assessment | Description | Length of scale/time needed to complete | Timing of administration |
| --- | --- | --- | --- |
| Pittsburgh Sleep Quality Index (PSQI)  [37-39] | Assesses sleep quality and disturbance over a 30-day time interval; 19 individual items generate seven “component” scores; score of 5 or higher indicative of poor sleep quality. Validated in populations with insomnia and other sleep disorders, psychiatric patients, and normal populations. Internal consistency and reliability coefficient ranging from 0.80 to 0.83 for its seven components. | 19 items (less than 5 minutes) | Day 28 of inpatient stay or just prior to discharge. |
| Epworth Sleepiness Scale (ESS)  [40-43] | A measure of general level of excessive daytime sleepiness over a one week time period; Individuals rate their usual chances of dozing off/falling asleep on a four point scale in 8 distinct activities common to everyday life and a score higher than 10 in indicative of excessive day time sleepiness. Has an Internal consistency and reliability coefficient between 0.70 and 0.88. | 8 items (2-3 minutes) | Within 7 days of discharge and 4-6 weeks post discharge. |
| Dysfunctional Beliefs and Attitudes about Sleep (brief version; DBAS-16)  [44] | This brief version of the original 30-item version, assesses sleep related cognitions including faulty beliefs and appraisals, unrealistic expectations, and perceptual and attention bias. Internal consistency is 0.77 for clinical and 0.79 for research samples. Average score of all items is used as the final score. | 16 items (less than 5 minutes) | Within 7 days of discharge and 4-6 weeks post discharge. |
| Self-Efficacy for Sleep Scale (SE-S)  [45-47] | Measures the level of confidence a person has in performing behaviors that might be helpful in initiating sleep. Scoring on a 5 point likert scale, the total score ranges from 9-45 with higher score indicative of greater confidence. Test-retest reliability has been established and the concurrent validity of scale was also established by comparison with PSQI, sleep diaries and objective sleep measures. Has an internal reliability ranging from 0.71 to 0.86. | 9 items (less than 5 minutes) | Within 7 days of discharge and 4-6 weeks post discharge. |
| Sleep-Related Behaviours Questionnaire (SRBQ)  [48] | This 32-item scale assesses the use of safety behaviors by individuals to promote sleep and cope with tiredness. Each item is scored on a 5-point Likert scale, with a possible score ranging from 0 to 128. The scale has discriminated between normal sleepers and those with insomnia in previous research and correlates with the PSQI (r = 0.78, p < .01). Each item on the scale also positively correlates with the Insomnia Severity Index (ISI), indicating that implementation of the techniques in the scale are associated with insomnia severity. | 32 items (less than 10 minutes) | Within 7 days of discharge and 4-6 weeks post discharge. |
| Penn Alcohol Craving Sale (PACS)  [32] | Measures alcohol craving using 5-item self-administered scale that asks about frequency, intensity, duration of thoughts about drinking along with ability to resist drinking and finally average rating of craving by the responder over the past week. Construct, predictive and discriminant validity has been established and it also has excellent internal consistency (Cronbach's alpha = 0.92). | 5 items (2-5 minutes) | Weekly throughout admission and 4-6 weeks post discharge. |
| Comprehensive Psychopathological Rating Scale (CPRS)  [34-36] | Assesses severity of psychiatric symptoms and observed behaviors. Nineteen self-assessed variables correspond to two subscales of CPRS ; Brief Scale for Anxiety (BSA) and Montgomery Asberg Depression Rating Scale (MADRS). BSA is a measure of pathological anxiety alone or in combination of other psychological or medical disorders. It consists of 10 items, all of which are rated on a 0-6 scale (0=no abnormality, 6=severe).  The MADRS, evaluating core symptoms of depression, is comprised of 10 items and is rated on a scale of 0-6. Nine items of this scale are based upon patient report, and one is on the rater’s observation during the interview. | 19 items (5-10 minutes) | Weekly throughout admission* |
| Clinical Institute Withdrawal Assessment – Alcohol revised (CIWA-Ar).  [33] | This10-item scale assesses the severity of alcohol withdrawal. The scale is a combination of patient reported and observation based responses and includes questions about nausea/vomiting, tremors, paroxysmal sweats, anxiety, agitation, tactile auditory and visual disturbances, headache, and orientation and clouding of sensorium. | 10 items | During the first few days of inpatient stay, CIWA scoring is done every 2-4 hours and as needed until the scores get consistently below the range of 5-7 |
